# Nut Allergy in Two Different Areas of Spain: Differences in Clinical and Molecular Pattern

**DOI:** 10.3390/nu9080909

**Published:** 2017-08-21

**Authors:** Elisa Haroun-Díaz, Julián Azofra, Eloína González-Mancebo, Manuel de las Heras, Carlos Pastor-Vargas, Vanesa Esteban, Mayte Villalba, Araceli Díaz-Perales, Javier Cuesta-Herranz

**Affiliations:** 1 Fundación IIS-Fundación Jiménez Díaz, 28040 Madrid, UAM, Spain; elisaharoun@hotmail.com (E.H.-D.); mheras@fjd.es (M.d.l.H.); cpastor@fjd.es (C.P.-V.); vesteban@fjd.es (V.E.); 2 Allergy Department, Hospital Central de Asturias, 33011 Oviedo, Spain; julian.azofra@sespa.es; 3 Unidad Alergia, Hospital Universitario de Fuenlabrada, 28942 Madrid, Spain; eloina.gonzalez@salud.madrid.org; 4 Facultad de Químicas, Universidad Complutense de Madrid, 28040 Madrid, Spain; maytevillalbadiaz@gmail.com; 5 Departamento de Biotecnología, ETS Ingenieros Agrónomos, 28040 Madrid, Spain; araceli.diaz@upm.es

**Keywords:** nut allergy, peanut, walnut, hazelnut, Bet v 1, LTP, Pru p 3, Phl p 12

## Abstract

Introduction: Different clinical and molecular patterns of food allergy have been reported in different areas of the world. The aim of the study is to evaluate differences in allergen patterns among nut-allergic patients in two different areas of Spain. Material and methods: A total of 77 patients with nut allergy from two different regions of Spain (Madrid and Asturias) were evaluated. Results: Hazelnut, peanut, and walnut were the three most frequent nuts eliciting allergy in both regions, but in a different order. Patients from Madrid experienced systemic reactions more often than patients from Asturias (73.5% Madrid vs. 50.0%, *p* < 0.05). The percentage of sensitizations to LTP (Lipid Transfer Protein) was higher than Bet v 1 (*p* < 0.05) in the Madrid area. The percentage of sensitizations in Asturias area was similar to LTP than Bet v 1 (Pru p 3 46.4%, Bet v 1 42.9%, ns). Bet v 1 was the predominant allergen involved among hazelnut-allergic patients (56.2%), while LTP was more common in peanut-allergic patients (61.5%). Conclusion: Walnut, hazelnut, and peanut were the most frequent nuts eliciting allergy in Spain. Despite this, important differences in molecular pattern were appreciated not only between both regions, but also among nut-allergic patients in Asturias. The different molecular pattern was linked to the frequency of systemic symptoms.

## 1. Introduction

Traditionally, the term anaphylaxis has referred to a systemic, immediate hypersensitivity reaction caused by IgE-mediated immunologic release of mediators from mast cells and basophils [1] and expressed by systemic symptoms (i.e., cutaneous, respiratory, gastrointestinal, or vascular symptoms). Food-induced anaphylaxis is a leading cause of anaphylaxis treated in emergency departments and hospitals around the world [2]. Nuts (including peanut and tree nuts) are one of the most common foods causing acute allergic reactions in children and adults, and nearly all nuts have been associated with fatal allergic reactions [3]. McWilliam et al. [3] evaluated the prevalence of tree nut allergy in different regions of the world by means of a systematic review. The results of the study proved that prevalence of individual nut allergies varied significantly by region, with hazelnut being the most common tree nut allergy in Europe, walnut and cashew in the USA, and Brazil nut, almond, and walnut most commonly reported in the UK. In addition, peanut allergy was the leading cause of death related to food-induced anaphylaxis in the United States [4].

Component resolved diagnosis allows the study of the molecules or allergens involved in the nut allergic reactions. Many allergens have been reported from nuts [5,6] and in many cases cross-reactivity has being found among them [7]. 

Although most nuts (i.e., almond, hazelnut, pine nut, walnut, peanut, etc.) belong to different botanical families without taxonomical relationships, cross-reactivity can occur due to shared homologue proteins, since most of the nut allergens belong to a small number of protein families sharing a 3-D structure, biologic function, and sequence identity to varying degrees. Along with that, different protein families have been associated to different risks of systemic reactions or anaphylaxis [8,9]. In this sense, different clinical and molecular patterns of food allergy have been reported in different areas of the world [10,11].

Nut allergy has been associated with Bet v 1 sensitization in Europe, and in Spain, nut allergy has been associated with LTP sensitization [10,12,13]. In this study we describe differences in nut allergy in two different areas of Spain in which allergy was clearly associated with different molecular patterns of sensitization and differing risks of systemic symptoms.

## 2. Material and Methods 

### 2.1. Study Population 

A total of 77 patients with nut allergy from two different regions of Spain participated in the study: Madrid (*n*: 49; Fundación Jiménez Díaz Hospital; Table 1 and Table 2) and Asturias (*n*: 28; Hospital Central de Asturias; Table 3 and Table 4). Inclusion criteria were patients diagnosed with nut allergy recruited during 2013–2016. Nut allergy was diagnosed in patients having a clear history of adverse reactions due to any nut (peanut, tree nut allergy, etc.) suggestive of IgE-mediated allergy, showing positive skin prick tests, or specific IgE and/or food challenge tests, following the diagnostic algorithm of the Food Adverse Reaction Committee of Sociedad Española de Alergia e Inmunología Clínica [14]. Patients suffering severe systemic reactions to nuts, as well as patients with typical, recent, repeated, and unequivocal reactions who had positive skin tests/specific IgE, did not undergo an oral challenge test to diagnose plant-food allergy [14].

Exclusion criteria were pregnancy or breastfeeding, extensive skin disease, serious psychiatric/psychological disturbances, contraindication to adrenaline treatment, alcohol or drug addiction, treatment with β-blockers, as well as any other condition which could either hamper protocol compliance or for which an oral challenge test is contraindicated [15]. 

### 2.2. Allergen-Specific IgE 

Allergen-specific IgE was measured with the ImmunoCAP System FEIA (ThermoFisher Scientific AB, Uppsala, Sweden) following the manufacturer’s recommendations. Venous blood samples were analyzed for IgE to nuts (peanut, hazelnut, almond, chestnut, pistachio, pine nut, and walnut), as well as to Pru p 3, Bet v 1, Phl p 12, Ara h 9, and Cor a 8.

### 2.3. Skin Prick-Prick Test

Skin prick tests were performed with a commercial battery (C.B.F. LETI, S.A; Tres Cantos, Spain) of nut extracts (almond, hazelnut, peanut, chestnut, sunflower seed, pine nut, walnut, pistachio) and prick-by-prick test with nuts (almond, hazelnut, peanut, chestnut, sunflower seed, pine nut, walnut, pistachio, and cashew), as well as a commercial battery (ALK-Abelló, Madrid, Spain) of pollen extracts, including *Lollium perenne*, *Betula verrucosa*, *Cupressus sempervirens*, *Platanus acerifolia*, *Artemisia vulgaris*, *Parietaria judaica, Salsola kali*, *Plantago lanceolata*, and *Olea europaea*. The ALK-Lancet needle (ALK-Lancet; ALK-Abelló, Horsholm, Denmark) was used for skin tests, which were performed according to EAACI guidelines [16]. Histamine phosphate at 10 mg/mL and normal saline solution were used as positive and negative controls, respectively. A weal with a diameter at least 3 mm larger than the negative control was considered a positive reaction. 

### 2.4. Ethical Consent 

The Fundación Jiménez Díaz Ethic Committee approved this study and written informed consent was obtained from all subjects. 

### 2.5. Statistical Analysis 

Statistical analysis was performed with SPSS (SPSS Inc., Chicago, IL, USA). The qualitative variables were expressed as a percentage (without taking into account the missing cases). For quantitative variables, means and standard deviation (SD) were calculated, and for specific IgE and SPT results, medians and 25th (Q1) and 75th (Q3) percentiles were given. A *Χ*^2^ test was used for comparisons of frequencies. Values were considered significant at a *p* value of less than 0.05.

## 3. Results

A total of 77 patients (Table 5) with nut allergy took part in the study: 49 patients from Madrid and 28 from Asturias. Patients from Madrid had a mean age of 30 years: 46.9% female (23 out of the 49 patients) and 53.1% male (26 out of the 49 patients). The mean age in Asturias was 33.4 years: 42.9% female (12 out of the 28 patients) and 57.1% male (16 out of the 28 patients). Fifteen patients younger than eighteen years participated in the study (eight in Madrid and seven in Asturias). 

The study results were focused on hazelnut, peanut and walnut because of they were the three most frequent nuts eliciting allergy in both regions. The most frequent in Madrid was walnut (32 patients—65.3%), followed by hazelnut (28 patients—57.1%), peanut (23 patients—46.9%), and almond (21 patients—42%); while in Asturias the most frequent nut was hazelnut (16 patients—7.1%), followed by walnut (14 patients—50.0%), peanut (13 patients—46.4%), and almond (three patients—10.7%). Other nuts elicited allergy less frequently.

Respiratory symptoms affected 81.6% of the patients in Madrid and 56.6% in Asturias. All patients from Madrid had associated asthma and rhinitis while in Asturias rhinitis was the most prevalent respiratory symptom (47% rhinitis, 29% rhinitis and asthma, and 23.5% asthma).

In Madrid, 77.5% of the patients were sensitized to grass pollen and 47% to birch pollen, but 44% of the patients were sensitized to both. In Asturias, 57.1% and 42.8% were sensitized to grass pollen and birch pollen, respectively, although only 3.6% were sensitized to both grass and birch pollens in Asturias.

Interestingly, 36 of the 49 nut allergic patients (73.5%) from Madrid had systemic symptoms, while only 14 of the 28 patients (50.0%) studied in Asturias experienced systemic reactions, the difference being statistically significant (*p* < 0.05). 26.5% patients from Madrid and 50% from Asturias had isolated OAS symptoms (*p* < 0.05). In both regions walnut was the nut that elicited systemic symptoms in a larger number of patients (22 patients in Madrid, seven patients in Asturias), while walnut elicited OAS in a larger number of patients from Madrid (10 patients) and hazelnut in Asturias (10 patients) (Table 6).

**Figure 1:**
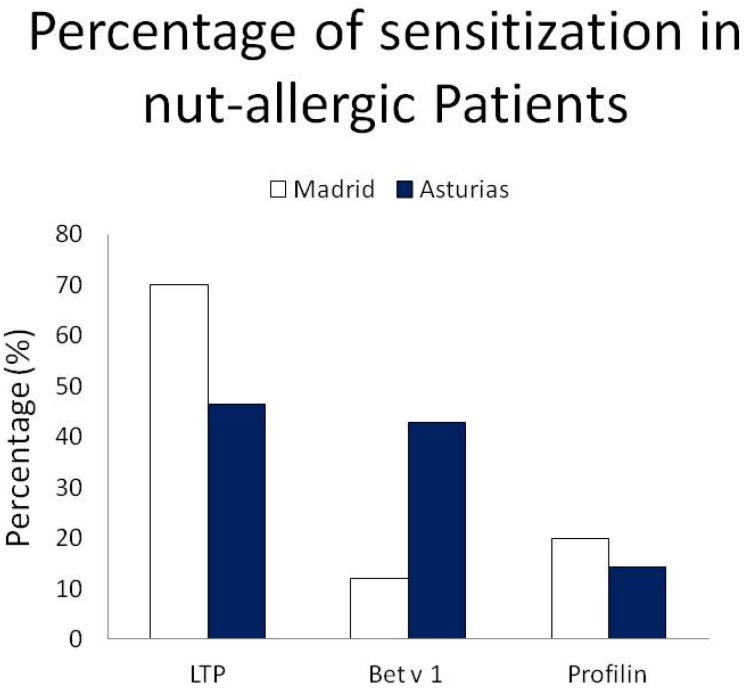
Differences in molecular pattern between Madrid and Asturias.

**Figure 2:**
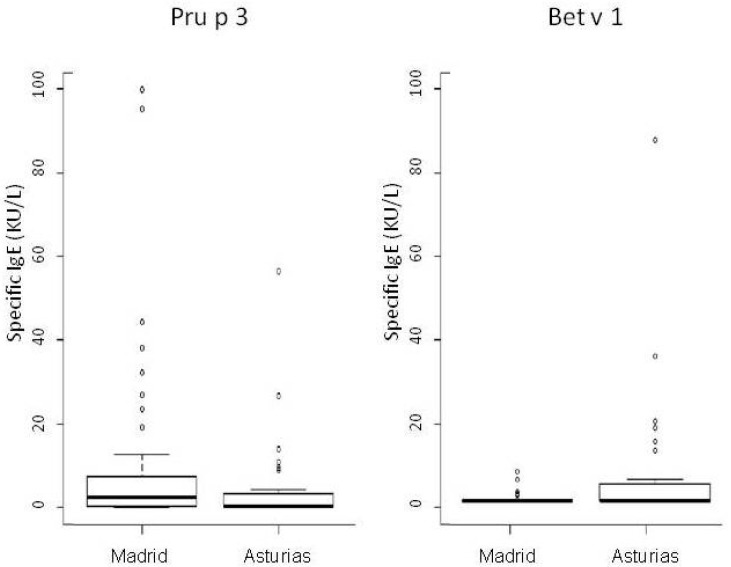
Specific IgE to LTP (Pru p 3) and Bet v 1 from both regions (median, Q1, and Q3).

On evaluating the pattern of sensitizations at the molecular level, we found important and interesting differences (Figure 1 and Figure 2 and Table 7). In Madrid, sensitization to Pru p 3 was 71.4% followed by Ara h 9 (61.2%), Cor a 8 (44.9%), Phl p 12 (20.4%), and Bet v 1 (12%). In the patient group from Asturias, 46.4% were sensitized to Pru p 3, 42.9% to Bet v 1, 33.3% to Ara h 9, 30% to Cor a 8, and 14.3% to Phl p 12.

**Figure 3:**
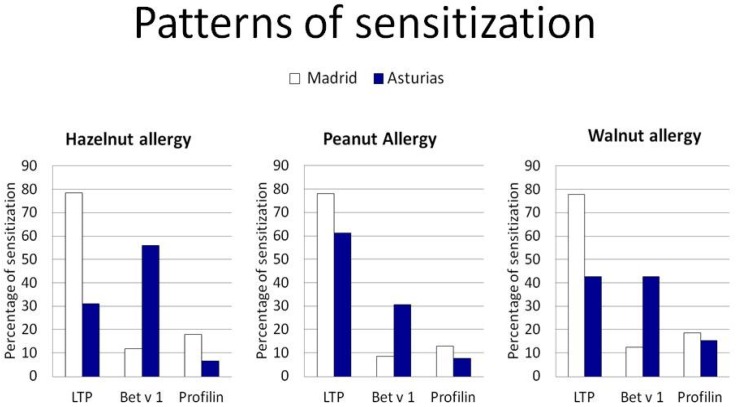
Differences in molecular pattern between Madrid and Asturias for patients allergic to hazelnut, peanut, or walnut.

In the Madrid region, the percentage of sensitizations to LTP was higher than sensitizations to Bet v 1 (LTP 71.4%; Bet v 1 12%; *p* < 0.05). This pattern of molecular sensitization was repeated in patients allergic to walnut (78.0% LTP vs. 12.5% Bet v 1), hazelnut (78.6% LTP vs. 12.0% Bet v 1), and peanut (78.6% LTP vs. 8.7% Bet v 1). Surprisingly, this situation was very different with respect to analyzing the molecular pattern of sensitization to different nut allergies in Asturias. Bet v 1 was the predominant allergen involved among patients allergic to hazelnuts (56.2% to Bet v 1 vs. 31.2% to LTP), while LTP was the predominant allergen among patients allergic to peanut (61.5% to LTP vs. 30.8% to Bet v 1). Sensitization to walnut-allergic patients was equivalent to LTP (42.9%) and Bet v 1 (42.9%) (Figure 3).

**Figure 4:**
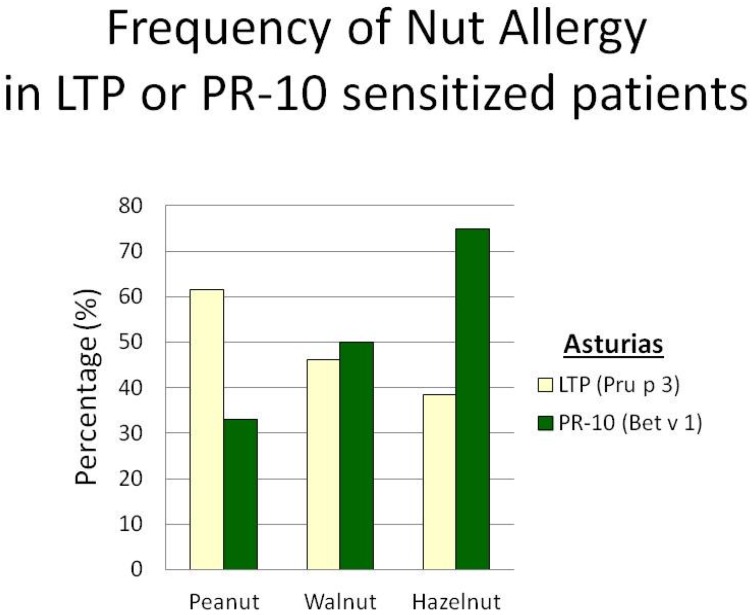
Frequency of nut allergies in patients sensitized to LTP and Bet v 1 from Asturias.

On evaluating the frequency of allergy to nuts in Asturias (Figure 4) among patients sensitized to LTP, peanut (61.5%) was the most frequent nut-eliciting allergy, followed by walnut (46.1%) and then hazelnut (38.5%). On evaluating the frequency of allergy to nuts among patients sensitized to Bet v 1, hazelnut (75%) was the most frequent nut-eliciting allergy, followed by walnut (50%) and then peanut (33%).

## 4. Discussion

This study found that walnut, hazelnut, and peanut were the most frequent nuts eliciting allergy in both regions of Spain. Despite this consistency, important differences in molecular pattern were seen not only between both regions, but also among different nuts in Asturias. The different molecular pattern was linked to the frequency of systemic symptoms suffered by patients.

Nut is a frequent cause of food allergy worldwide and is responsible for severe and near-fatal anaphylactic reactions [2]. Spain is probably one of the most typical examples of plant food allergy due to LTP sensitizations [10,11,12,13,17,18]. 

In this study we have evaluated the most frequent nut-eliciting allergy in two different regions from Spain. While, a distance of only 400 km separate them, Madrid has a continental climate and Asturias a maritime climate. An interesting result to be emphasized was that percentage of positive skin prick test results to birch pollen was similar in both regions (47% in Madrid and 42.8% in Asturias) without statistically significant differences. Curiously, on evaluating primary sensitizations, 12% of the patients from Madrid were sensitized to Bet v 1, while 42.8% were sensitized in Asturias, the difference being now statistically significant (*p* < 0.05). In this sense, we should remember that Madrid is an area with absence or low atmospheric level of birch tree pollen. The considerable percentage of patients sensitized to birch pollen in the Madrid area with a low percentage of sensitization to Bet v 1 have been previously reported [17].

Walnut, peanut, and hazelnut were the most frequent nuts eliciting allergy in both regions of Spain and, as a result, the study was focused on the analysis of differences of these three nuts. Walnut was more frequent in Madrid, but there were small differences among the tree nut prevalence in Asturias, with hazelnut being more frequent in this case but without significant statistical differences. These results were different from those reported by McWilliam et al., who analyzed the prevalence of nut allergy worldwide [3]. These differences might be explained by the allergen molecule involved in the allergic reactions, since, as previously stated, LTP was usually the major allergen involved in food allergy in Spain [10,12,13,18].

Another important result to be emphasized was the allergen pattern involved in the allergic reactions. On the one hand, there was a predominant pattern of LTP sensitization in the Madrid region, which was in accordance with previously-reported studies [10,12,13,18]. This pattern was similar to the evaluated individual nuts (i.e., walnut, hazelnut and peanut). On the other hand, a mix of LTP and Bet v 1 sensitizations highlighted the pattern in Asturias region, however, the pattern changed depending on the nut evaluated. A predominant pattern of LTP was found in peanut allergy, Bet v 1 pattern in hazelnut allergy, and a mixed pattern (LTP and Bet v 1) to walnut allergy. These data are especially relevant because, to the best of our knowledge, this is the first time in which the predominant allergen family eliciting allergy in the same area changed from one nut to another. Nonetheless, we are conscious that it should be confirmed in studies with a higher number of patients.

Results of this report, along with other nut allergy studies from Spain [10,11,12,13,17,18], indicate that three different patterns of nut allergy coexist in Spain: LTP pattern, the most frequent pattern in most places for older children (>5 years old) and adult nut allergic patients; Bet v 1 pattern, which was significant in areas with patients sensitized to Bet v 1 dominated by hazelnut allergy; and, finally, storage protein pattern (2S albumin) in nut allergic children (<5 years old). 

## 5. Conclusions

In this study we found that walnut, hazelnut, and peanut were the most frequent nuts eliciting allergy in both regions of Spain. Despite this, important differences in molecular pattern were appreciated not only between both regions, but also among nuts in Asturias. The different molecular pattern was linked to the frequency of anaphylaxis.

## Figures and Tables

**Table 1:** Nut allergic patients from Madrid.

No.	Age	Sex	Specific IgE					
			Pru p 3	Phl p 12	Bet v 1	Peanut	Hazelnut	Walnut
1	37 years old	F	6.02	ND	0.00	1.17	2.88	5.09
2	20 years old	M	0.42	0.40	0.00	0.96	2.79	0.25
3	50 years old	M	4.00	0.18	0.02	1.37	0.94	2.94
4	26 years old	F	0.01	0.00	0.00	0.04	0.02	0.00
5	22 years old	M	19.20	0.08	0.01	4.65	3.22	12.30
6	20 years old	F	9.35	0.00	0.00	2.42	0.85	4.77
7	20 years old	M	0.02	15.70	0.01	8.84	0.69	1.88
8	23 years old	M	4.35	0.00	0.00	3.19	0.72	3.01
9	45 years old	M	38.30	ND	ND	11.1	1.77	13.9
10	21 years old	F	5.75	0.00	0.00	1.97	1.17	2.66
11	22 years old	M	1.40	ND	2.06	0.73	0.62	8.81
12	27 years old	M	1.11	0.47	0.00	0.47	0.00	0.42
13	12 years old	M	1.11	0.47	0.00	0.47	0.00	0.42
14	34 years old	M	7.83	ND	1.67	4.14	0.78	0.65
15	40 years old	M	1.26	0.01	0.00	0.65	0.39	1.08
16	32 years old	F	4.12	0.16	0.02	1.78	1.36	2.67
17	29 years old	M	1.79	0.02	0.02	0.62	0.28	0.67
18	40 years old	M	2.40	0.02	0.01	2.31	0.85	3.12
19	32 years old	F	12.60	0.03	ND	5.73	4.09	10.9
20	32 years old	M	0.11	0.02	ND	0.13	0.07	0.08
21	25 years old	F	0.22	20.20	ND	20.8	1.19	1.87
22	29 years old	M	27.10	0.05	ND	9.58	5.59	18.8
23	25 years old	F	3.02	0.00	6.84	0.41	16.6	1.95
24	60 years old	F	32.30	0.10	ND	5.44	2.15	19.6
25	28 years old	F	0.72	0.03	0.01	1.22	0.14	0.52
26	39 years old	F	0.01	6.01	0.00	3.22	0.54	0.87
27	44 years old	F	1.20	0.66	0.00	0.93	0.65	1.28
28	38 years old	F	0.01	0.00	0.00	0.00	0.09	0.00
29	17 years old	F	0.23	0.02	0.03	0.04	0.84	5.84
30	16 years old	M	3.90	0.34	0.00	1.95	0.98	3.13
31	39 years old	F	0.27	0.05	0.00	0.12	0.05	0.14
32	40 years old	F	2.86	0.01	0.00	1.23	0.12	2.03
33	4 years old	M	0.04	ND	0.00	0.03	0.13	ND
34	12 years old	F	0.03	0.01	0.04	100	0.17	2.24
35	26 years old	M	23.70	0.10	0.10	3.00	11.30	19.9
36	40 years old	M	100.00	0.22	0.20	33.3	22.60	59.1
37	66 years old	F	7.58	0.00	0.00	2.20	2.79	6.76
38	3 years old	M	12.90	ND	ND	19.5	ND	5.73
39	53 years old	F	1.00	0.09	1.25	0.03	0.61	0.01
40	33 years old	M	5.87	8.79	5.09	8.47	3.54	6.17
41	7 years old	M	0.19	0.02	0.06	1.82	0.85	ND
42	39 years old	M	3.34	0.00	2.21	3.17	6.18	4.99
43	52 years old	F	0.04	1.35	0.01	0.56	0.21	0.19
44	29 years old	F	0.15	5.96	0.08	3.64	1.41	1.52
45	25 years old	M	2.03	0.00	0.01	0.12	0.18	0.87
46	29 years old	F	44.40	0.00	0.00	24.20	14.5	33.6
47	29 years old	M	95.50	0.07	0.04	1.95	3.36	3.28
48	13 years old	F	0.00	0.03	0.00	100.00	2.08	3.11
49	31 years old	M	3.18	0.30	0.01	2.01	1.66	2.61

F: Female; M: Male; ND: Not done.

**Table 2:** Nut allergic patients from Madrid.

No.	Specific IgE					
	Ara h 9	Cor a 8	Almond	Chestnut	Pistachio	Pinenut
1	1.42	3.51	0.93	0.78	0.03	0.14
2	0.53	3.18	0.38	0.26	0.18	0.12
3	1.81	2.58	0.25	1.26	0.05	0.11
4	0	0	0.02	0.05	0.02	0.03
5	14.6	8.85	2.22	1.45	2.14	0.11
6	3.2	2.89	0.81	0.05	0.06	0
7	0.01	0	0.37	4.48	0.67	0.92
8	3.33	1.59	0.73	1.1	0.04	0.32
9	ND	ND	5.34	3.31	0	ND
10	1.46	ND	1.04	1.58	0	0
11	ND	ND	0.46	0.81	2.2	0
12	0.39	0	0	0	0	0
13	0.39	0	0	0	0	0
14	4.43	ND	2.01	1.42	0.94	ND
15	0.76	0.46	0.22	0.28	0.02	0
16	1.05	2.81	0.56	0.58	0.13	0.03
17	0.29	0.1	0.3	0.5	0.41	0.3
18	2.35	0.46	1.88	1.91	0.62	ND
19	7.38	4.2	4.28	4.07	0.51	0.06
20	0.05	0.01	0.12	0.1	0.07	0.83
21	0	0	0.65	8.73	1.32	4.46
22	18.6	11.5	2.73	4.26	0.38	2.18
23	0.39	0.39	0.42	0	0	0
24	18.1	16	0.56	6.71	0.24	0.12
25	0.43	0.22	0.14	0.2	0.04	0.07
26	0	0	0.64	3.15	0.71	1.68
27	1.27	0.67	0.28	0.5	0.13	0.28
28	0.01	0	0.03	0.02	0.01	0.03
29	0.09	0	0.02	0.14	0.04	0.02
30	3.2	1.98	0.9	1.77	0.14	0.09
31	0.13	0.07	0.04	0.06	0.1	0
32	4.16	0.03	0.11	0.79	0.03	0.03
33	0	0	0.02	0.05	85.6	0
34	0	0	0.5	0.08	2.08	0.05
35	17.6	13.9	1.75	1.7	0.87	0.51
36	100	46.8	15.8	12.8	7.35	3
37	6.58	5.93	1.01	1	0.08	0
38	11.3	ND	7.72	ND	0.85	ND
39	0	0	0.4	0.21	0.1	0.01
40	0.53	0.12	3.56	8.47	1.58	1.74
41	0.01	0	0.13	1.88	0.84	0.65
42	5.56	1.46	1.28	1.83	0.63	0.47
43	0.02	0	3.72	0.53	0.76	0.12
44	0.07	0.07	1.76	3.03	2.21	1.97
45	0.16	0.21	0.12	0.03	0.31	0.01
46	40.6	15.5	5.93	13.3	1.18	1.83
47	44.4	3.64	4.6	4.56	1.8	0.75
48	0	0	3	0.12	1.6	0.11
49	1.86	1.87	1.38	1.7	0.7	0.31

F: Female; M: Male; ND: Not done.

**Table 3:** Nut allergic patients from Asturias.

No.	Age	Sex	Specific IgE					
			Pru p 3	Phl p 12	Bet v 1	Peanut	Hazelnut	Walnut
1	58 years old	F	0.00	0.00	4.99	0.26	2.50	0.00
2	79 years old	F	0.06	ND	ND	0.06	0.04	ND
3	42 years old	F	0.09	0.04	85.40	0.37	57.9	0.04
4	35 years old	F	0.01	0.00	14.00	0.01	4.74	0.00
5	39 years old	M	1.09	0.02	0.92	0.31	0.66	0.42
6	30 years old	M	0.54	0.49	3.12	2.30	2.07	0.69
7	14 years old	M	0.03	0.01	0.00	0.02	0.04	1.22
8	6 years old	M	0.02	0.02	0.00	1.33	0.81	48.40
9	6 years old	M	0.53	0.03	0.03	0.32	22.5	7.36
10	29 years old	M	56.5	0.11	0.06	8.41	14.8	38.8
11	8 years old	F	0.15	0.11	0.14	0.54	1.48	3.69
12	27 years old	M	9.07	0.06	0.03	1.66	1.90	5.06
13	24 years old	F	0	0.04	1.87	0.53	1.81	0.04
14	41 years old	M	1.76	0.01	0.00	1.13	1.08	1.69
15	44 years old	M	9.66	0.01	0.01	0.25	0.58	0.84
16	37 years old	M	0.3	0.00	00.00	0.02	0.05	0.06
17	30 years old	M	26.8	0.06	0.03	4.66	3.79	5.15
18	16 years old	M	0.04	0.03	34.4	8.63	24.50	0.09
19	40 years old	F	2.64	0.00	2.46	0.07	1.18	0.29
20	31 years old	M	11	0.04	0.06	4.48	5.36	4.95
21	16 years old	M	0.05	0.03	0.02	16.2	0.04	1.50
22	12 years old	F	0.13	0.81	17.40	4.29	9.54	2.85
23	61 years old	F	0.03	0.61	12.00	0.55	5.09	0.07
24	51 years old	M	0.17	3.21	18.90	2.14	7.69	0.19
25	47 years old	F	4.54	0.00	0.00	1.30	0.52	3.17
26	33 years old	F	14	0.00	0.00	3.06	2.31	8.27
27	45 years old	F	0	0.00	0.82	0.19	0.77	0.02
28	33 years old	M	1.62	0.00	0.00	0.30	0.02	1.03

F: Female; M: Male; ND: Not done.

**Table 4:** Nut allergic patients from Asturias.

No.	Specific IgE					
	Ara h 9	Cor a 8	Almond	Chestnut	Pistachio	Pinenut
1	0	0	0.21	0.73	0.01	0
2	0	0	0.83	0.06	0.14	0.06
3	0.06	0	2.02	10.1	0.04	0.02
4	0	0	0.19	0.66	0.01	0
5	0.33	0.25	0.19	0.41	0.03	0.13
6	0.12	0.08	0.1	1.12	2.12	0.18
7	0	0	0.01	0.04	0.02	0.01
8	0	0	0.1	0.16	0.15	0.01
9	0.13	8.83	0.26	2.91	0.28	0.4
10	27.1	12.1	7.98	5.46	0.91	3.19
11	0.06	0.04	0.43	0.57	0.45	0.44
12	3.43	1.26	0.85	3.02	1.66	0.62
13	ND	0	0.03	0.45	0.06	ND
14	1.62	0.94	0.34	1.35	0.1	0.05
15	0.69	0.22	0.49	0.19	0.08	0.02
16	0.06	0.13	0	0.03	0.02	0
17	8.76	1.36	1.09	1.09	0.17	0.1
18	0.05	0.02	1.6	6.95	0.11	0.1
19	5.44	0.77	0.93	0.2	0.11	0
20	4.71	9.38	1.1	1.39	0.34	0.09
21	0	0	0.21	0.1	0.03	0.02
22	0.08	0	0.29	2.28	5.65	0.07
23	0	0	0.35	0.4	0.18	0.07
24	0	0.07	0.1	0.79	0.09	0.18
25	2.25	1.23	0.27	0.07	0.14	0.99
26	7.54	5.99	2.9	2.51	0.15	0.05
27	0	0	0	0.17	0.16	0
28	1.31	0	0.05	0.5	0.08	0

F: Female; M: Male; ND: Not done.

**Table 5:** General characteristics of nut allergic patients.

Characteristics	Madrid	Asturias
Nut allergic patients	49	28
Age (mean ± SD)	30.1 ± 13.5	33.4 ± 17.4
Sex	46.9% ♀/53.1% ♂	42.9% ♀/57.1% ♂
Nut Allergy		
Walnut	32 (65.5%)	14 (50.0%)
Hazelnut	28 (57.0%)	16 (57.1%)
Peanut	23 (46.9%)	13 (46.4%)
Almond	21 (42.0%)	3 (10.7%)
Symptoms		
OAS	13 (25.6%)	14 (50.0%)
SS	36 (73.5%)	14 (50.0%)

SD: standard deviation; OAS: Oral Allergy Syndrome; SS: Systemic Symptoms.

**Table 6:** Symptoms to nuts in patients from Madrid and Asturias.

Symptoms	Madrid		Asturias	
	*n*	%	*n*	%
Hazelnut	28		16	
OAS	9	32.1%	10	62.5%
SS	19	67.9%	6	37.5%
Peanut	23		13	
OAS	5	21.7%	8	61.5%
SS	18	78.3%	5	38.5%
Walnut	32		14	
OAS	10	31.2%	7	50.0%
SS	22	68.8%	7	50.0%

**Table 7:** Differences in specific IgE levels to purified allergens between Madrid and Asturias (Median, Q1, Q3).

Allergen	Asturias	Madrid	*p*
Pru p 3	0.23 (0.04, 3.11)	2.40 (0.23, 7.58)	0.037
Phl p 12	0.03 (0.00, 0.06)	0.05 (0.01, 0.32)	0.205
Bet v 1	0.06 (0.00, 4.05)	0.01 (0.00, 0.04)	0.005
